# Metal Oxide Nanoparticle Based Electrochemical Sensor for Total Antioxidant Capacity (TAC) Detection in Wine Samples

**DOI:** 10.3390/bios8040108

**Published:** 2018-11-14

**Authors:** Cristina Tortolini, Paolo Bollella, Rosaceleste Zumpano, Gabriele Favero, Franco Mazzei, Riccarda Antiochia

**Affiliations:** Department of Chemistry and Drug Technologies, Sapienza University of Rome—P.le Aldo Moro 5, 00185 Rome, Italy; cristina.tortolini@uniroma1.it (C.T.); paolo.bollella@uniroma1.it (P.B.); rosaceleste.zumpano@uniroma1.it (R.Z.); gabriele.favero@uniroma1.it (G.F.); franco.mazzei@uniroma1.it (F.M.)

**Keywords:** total antioxidant capacity, antioxidants, nanoceria, enzyme mimetic, SPE sensor, disposable sensor, wine

## Abstract

A single-use electrochemical screen-printed electrode is reported based on biomimetic properties of nanoceria particles (CeNPs). The developed tool showed an easy approach compared to the classical spectrophotometric methods reported in literature in terms of ease of use, cost, portability, and unnecessary secondary reagents. The sensor allowed the detection of the total antioxidant capacity (TAC) in wine samples. The sensor has been optimized and characterized electrochemically and then tested with antioxidant compounds occurred in wine samples. The electrochemical CeNPs modified sensor has been used for detection of TAC in white and red commercial wines and the data compared to the 2,2′-azino-bis(3-ethylbenzthiazoline-6-sulphonic acid (ABTS)-based spectrophotometric method. Finally, the obtained results have demonstrated that the proposed sensor was suitable for the simple and quick evaluation of TAC in beverage samples.

## 1. Introduction

Antioxidant capacity is an ability of organisms or food to catch free radicals and prevent their harmful effects. Substances with antioxidative properties are called antioxidants and have received much attention in recent years. They have ability to fight against the oxidative processes [1], to promote health and to prevent a wide variety of diseases: atherosclerosis, type 2 diabetes, neurodegeneration (Alzheimer’s and Parkinson’s diseases) [2,3] and cancer [4]. Epidemiological studies recommend to introduce in the diet substances as fruits, vegetables and less processed staple foods ensure the best protection against possible diseases caused by oxidative stress, such as coronary heart disease, obesity, hypertension, and cataracts [5]. The explanation consists in the beneficial health effect, due to antioxidants present in fruit and vegetables [6]. In particular, polyphenols are naturally-occurring antioxidants found widely in the fruits, vegetables, cereals, dry legumes, chocolate and beverages, such as tea, coffee, or wine. Polyphenols and other food phenolics are the subject of increasing scientific interest because offer a great hope for the prevention of human diseases [7].

Total antioxidant capacity (TAC) is the measure of the quantity of free radicals scavenged by a test solution [8], for evaluating the antioxidant capacity in samples of different nature. Several methods have been proposed for the determination of the TAC of body fluids [9,10,11,12,13], of biological samples [14,15], and food extracts [16,17]. Usually, the antioxidant capacity is given as the Trolox (6-hydroxy-2,5,7,8-tetramethylchroman-2-carboxylic acid) equivalent antioxidant capacity (TEAC) [18,19], as the ferric reducing antioxidant power (FRAP) [20,21], as the cupric reducing antioxidant capacity (CUPRAC) [22,23,24], as the oxygen radical absorption capacity method (ORAC) [25,26], as the 2,2′-azino-bis(3-ethylbenzthiazoline-6-sulphonic acid (ABTS) [25], and as the DPPH (2,2-diphenyl-1-picrylhydrazyl) method [27], respectively based on the different spectrophotometric methods which have been used to estimated it.

Recently, also electrochemical methods have greatly contributed to measure of TAC based on biosensors [28,29,30,31] and sensors [32,33]. They offer sensitivity, inexpensive instrumentation, fast response, small volumes [34,35,36,37].

In the last years, the application of nanomaterials resulted in many advantages for the sensing systems, including the observation of enhanced electrocatalytic phenomena with benefits in chemical and biosensing and improving analytical performance of classical sensing platforms [37,38,39,40,41,42,43,44,45,46]. Some authors reported nanomaterial based sensor for determination of polyphenols in foodstuffs [47,48,49,50,51,52,53,54,55].

Different kinds of nanomaterials have been used as stable and low-cost alternatives to biomolecules in (bio)analytical methods. The materials comprise metal/metal oxides, metal complexes, nanocomposites, porphyrins, phthalocyanines, smart polymers, and carbon nanomaterials [56,57]. Cerium oxide nanoparticles (CeNPs) or nanoceria particles have attractive catalytic and electrochemical features [58]. Moreover, ceria is an excellent co-immobilization material for a variety of oxidase and peroxidase enzymes such as horseradish peroxidase [59,60], glutamate oxidase [61]. Its catalytic activity can be exploited to develop highly sensitive, enzymeless H_2_O_2_ sensors [62] and for the fabrication of third generation biosensors [63]. Ceria has high oxygen mobility at its surface [64,65] and a large oxygen diffusion coefficient, which facilitates the conversion between valance states Ce^4+^/Ce^3+^ [66] that allow oxygen to be released or stored in its crystalline structure [67,68].

Recently, CeNPs have also been reported to have multienzyme, including superoxide oxidase, catalase and oxidase, and mimetic properties [69,70,71], and have emerged as a fascinating material in biological fields, such as in bioanalysis [72,73,74], biomedicine [75,76,77], and drug delivery [78,79]. Catalytically active nanoceria offer several advantages over natural enzymes, such as controlled synthesis at low cost, tunable catalytic activities and high stability against severe physiological conditions [80].

In the present work, we developed an electrochemical disposable sensor modified with nanoceria particles for determination of total antioxidant capacity (TAC) in real samples. Firstly, the sensor was characterized electrochemically and then has been tested for some common antioxidants present in wines, such as gallic acid, caffeic acid, ascorbic acid, quercetin, *trans*-resveratrol and, finally, for the detection of TAC in six white and red commercial wine samples. The results relative to wine samples obtained with the electrochemical method were compared to those carried out with the spectrophotometric method (ABTS-based method). The modification procedure was demonstrated to be very simple and the developed sensor resulted to be easy-to-use, robust and cheap without involving labeled reagents.

## 2. Materials and Methods

### 2.1. Chemicals and Reagents

All chemicals used were analytical grade and were used as received without any further purification. In particular: sodium monobasic phosphate (Na_2_HPO_4_), sodium dibasic phosphate NaH_2_PO_4_, potassium chloride (KCl), potassium ferricyanide (III) (K_3_[Fe(CN)_6_]), cerium (IV) oxide NPs (20 wt% colloidal dispersion in acetic acid 2.5 wt%, d = 30–60 nm), gallic acid (GA), caffeic acid (CA), quercetin (Q), *trans*-resveratrol (t-R), ascorbic acid (AA), and dimethyl sulfoxide were purchased from Sigma-Aldrich (Buchs, Switzerland). For the TEAC assay: 2,2′-azino-bis(3-ethylbenzothiazoline-6-sulphonic acid (ABTS) and potassium persulfate (K_2_S_2_O_8_) were also supplied by Sigma-Aldrich (Buchs, Switzerland).

All the solutions were prepared in phosphate buffer 0.1 M, KCl 0.1 M, pH 7.4 (PBS buffer) high-purity deionized water (resistance: 18.2 MΩ × cm at 25 °C; TOC < 10 µg L^−1^) obtained from Millipore (Molsheim, France) has been used to prepare all the solutions.

A solution of 1.1 mM K_3_[Fe(CN)_6_] in PBS buffer was used in cyclic voltammetric experiments for determination of electroactive area using the Randles–Ševćik equation. Stock solutions of 10 mM of several phenolic (GA, CA, Q and t-R) and not phenolic antioxidants (AA) were prepared daily before use: all substances in PBS buffer only the quercitin had need of few drops of DMSO.

The value of pH 7.4 was chosen because from early studies in literature was reported that at this value the particles have the highest oxidase-like activity against phenolic compounds [81]. Moreover, all the experiments were carried out at room temperature, approximately 25 °C.

### 2.2. Electrochemical Measurements

Electrochemical measurements were performed using a portable PalmSens potentiostat (PalmSens, Houten, The Netherlands) controlled by means of the PSTrace 4 program (Vers. 4.4 PalmSens BV). All the experiments were conducted using a three screen-printed electrodes system from Orion High Technologies S.L. (Parla, Madrid, Spain). In particular: Nanostructured carbon (OHT-000), carboxylic acid functionalized multi-walled carbon nanotubes (OHT-069) and Carboxylic acid functionalized multi-walled carbon nanotubes-Fe_3_O_4_ superparamagnetic nanoparticles (OHT-102) screen printed electrodes, respectively. The working electrodes were different for each sensor (with a surface diameter of 4 mm) but the counter electrode (graphite) and the reference one (Ag/AgCl) were the same for all the SPEs.

All absorbance measurements were made at the specified wavelength (731 nm) of the selected spectrophotometric method (ABTS-based method) using a T60U Spectrometer PG Instruments Ltd. (Wibtoft Leicestershire, Lutterworth, UK).

### 2.3. Electrochemical Method

The procedure of square wave voltammetry (SWV) was carried out as analytical technique to develop the calibration curve of GA used as standard and to test the selected wine samples and for the other antioxidant compounds. The frequency (f) and pulse amplitude at 25 Hz and 50 mV, respectively. The OHT-069 modified SPE was used as sensor.

### 2.4. Sensor Modification by Using CeNPs

The surface modification procedure was realized by two steps: first, a colloidal NPs suspension of 2% (*w*/*v*) nanoceria was prepared by dispersing particles in distilled water, then 5 µL of the solution were dropped onto the working electrode surface of the SPE and let it dry for two days at room temperature until use. Electrodes were stored at room temperature.

### 2.5. ABTS-Based Method

The ABTS antioxidant assay was slightly modified and carried out as described in literature [82]. Briefly, 7 mM ABTS solution prepared in 2.5 mM K_2_S_2_O_8_ was incubated in the dark for about 15 h at room temperature to generate ABTS radicals. Then, the solution was diluted 400 times with distilled water. The white wines and the red ones were diluted 10 and 100 times respectively with distilled water. Next 100 µL of each sample were mixed with 2.5 mL of ABTS radical solution and 0.4 mL H_2_O and the absorbance has read after 3 min at 731 nm. The phenolic antioxidant gallic acid (GA) was used as standard, and triplicates were analysed for each sample.

## 3. Results and Discussion

### 3.1. Electrochemical Characterization

#### 3.1.1. Comparison of Electrochemical Performances before and after CeNPs Sensor Modification

Firstly, the improvement of the electrochemical performances by the simple modification of the electrode surface using CeNPs has been demonstrated by calculating the electroactive area (A_e_) of three different SPE sensors.

The A_e_ for the electrodes were obtained by cyclic voltammetry (CV) using a solution of 1.1 mM K_3_[Fe(CN)_6_] in PBS buffer solution as probe at different scan rates. For a reversible process, the Randles–Ševćik equation has been used:

I_p_ = 2.686 10^5^ n ^3/2^ A_e_ D_0_^1/2^ C_0_ v^1/2^

where I_p_ is peak current in ampere (A), n is number of electrons transferred of K_3_[Fe(CN)_6_] by CV in the redox event (usually 1), A_e_ is electroactive area (cm^2^), D_0_ is diffusion coefficient (7.6 × 10^−6^ cm^2^ s^−1^), C_0_ is the concentration (mol cm^−3^), and v is the scan rate (Vs^−1^) [83]. By using the slope of the plot of I_p_ vs v^1/2^, the electroactive areas were calculated and the results are shown in Table 1. In bare OHT-000, OHT-102, and OHT-069 SPEs, the electrode surface was found to be: 2.42, 6.26, and 8.50 mm^2^ respectively. For the same modified SPEs the surface was nearly 7, 3, and 2.5 times greater. The presence of the MWCNTs in both OHT-102 and OHT-069 bare sensors resulted in a higher A_e_ value in comparison to OHT-000 bare sensor, due to the well-known peculiarities of nanomaterials (e.g., large surface-to-volume ratios, their physicochemical properties, composition, and shape and their robustness, etc.) [39,40]. Moreover, the modification procedure of the different working electrode surface by the presence of cerium nanoparticles increased quite notable the peak current signal and consequently the surface activity. This was attributed to their capability to facilitate the electron transfer [60] and to improve the biocatalytic signals [84].

**Figure 1:**
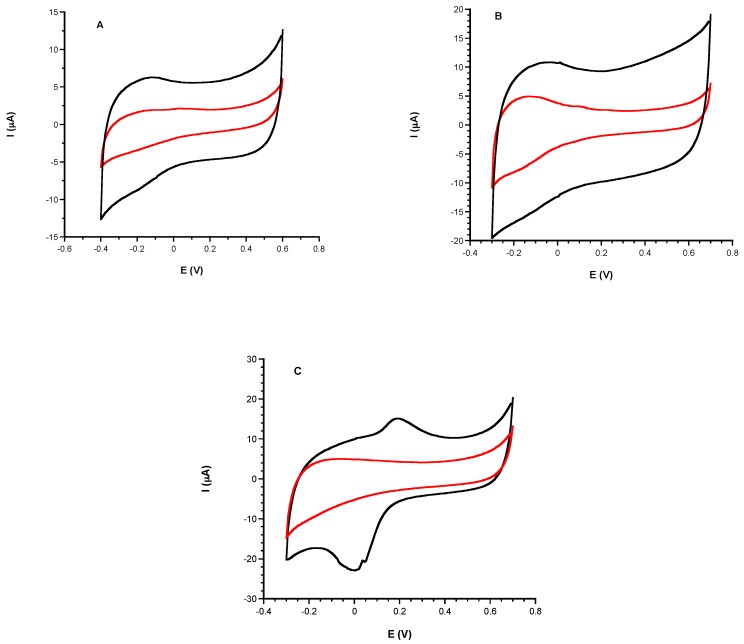
CVs of OHT-000 (**A**), OHT-069 (**B**), and OHT-102 (**C**) bare sensors (red line) and after modification by CeNPs (black line) in PBS buffer solution, ν = 100 mV s^−1^.

**Figure 2:**
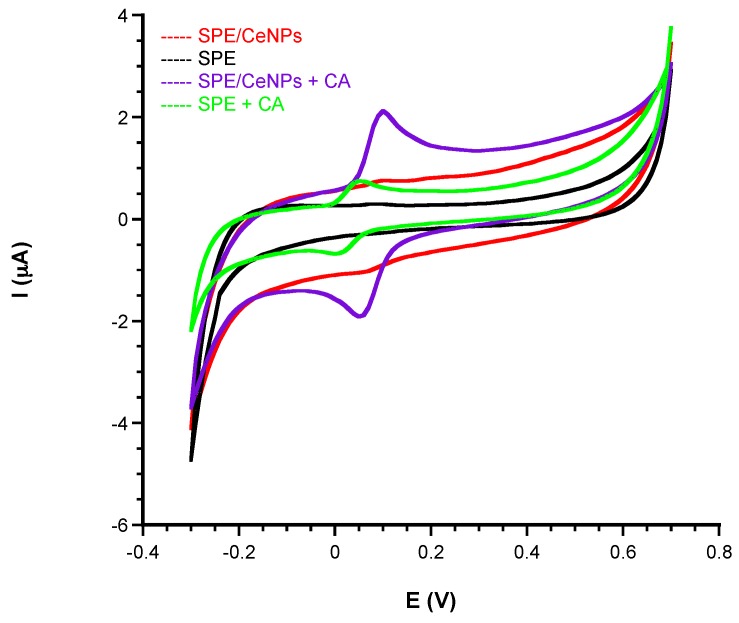
CVs of OHT-069 bare SPE (black line) and modified SPE by CeNPs (red line) in PBS buffer solution and the same sensors OHT-069 bare SPE (green line) and modified SPE by CeNPs (blue line) in the presence of 0.8 μM di CA at ν = 10 mV s^−1^.

In addition, the comparison was also observed in function of PBS buffer solution (Figure 1) and one of the selected antioxidants, the caffeic acid (Figure 2). The modified electrode had no electrochemical activity in phosphate buffer solution (Figure 1A,B, black lines) and showed a similar CV profile to the bare electrodes, respectively (Figure 1A,B, red lines). Conversely, for the OHT-102 a couple of peaks was observed (Figure 1C, black line), probably due to Fe_3_O_4_ nanoparticles redox behaviour, which are directly reduced at the electrode surface according to the following reaction:

(1) Fe_3_O_4_ (s) + 2 e^−^ + 6H^+^ (aq) → 2Fe^2+^ (aq) + 3H_2_O + FeO (s)

Then, Fe^2+^ ions produced could combine with phosphate ions in the buffer solution:

(2) Fe^2+^ (aq) + HPO_4_^2−^ (aq) → FePO_4_(s) + e^−^ + H^+^ (aq)

Here after, the solid FePO_4_ at the surface of electrode could be responsible for observed redox behaviour [85,86]. For all the three modified sensors the background current becomes larger, due to the CeNPs can increase the surface activity remarkably. The CeNPs-induced oxidation of CA was studied in the potential range between −0.4 and 0 V, as example in Figure 2 is reported the electrochemical performance of the OHT-069 SPE sensor. The CVs of bare and modified SPE carried out in PBS buffer solution showed a similar voltammogram, but in the case of the CeNPs modified sensor the area was broader and it showed no peaks in PBS indicating lack of electrochemical activity of these cerium particles in the potential range tested (Figure 2, red line). Observing the electrochemical behaviour of the bare OHT-069 SPE (Figure 2, green line) and the modified one (Figure 2, blue line) in the presence of 0.8 μM CA solution a similar profile is recorded. Anodic and catodic peaks were observed in both cases. In the CV of the CeNPs/SPE the peaks are wider and shifted to higher potential values compared to those observed for the bare/SPE. In identical experimental conditions, sensors in presence of nanoparticles showed a more evident response in the same potential range comparing to bare carbon electrode. This difference demonstrates the role of CeNPs to catalytically form reducible quinones onto the electrode surface and, moreover, to be better able to quantitatively detect antioxidants.

**Figure 3:**
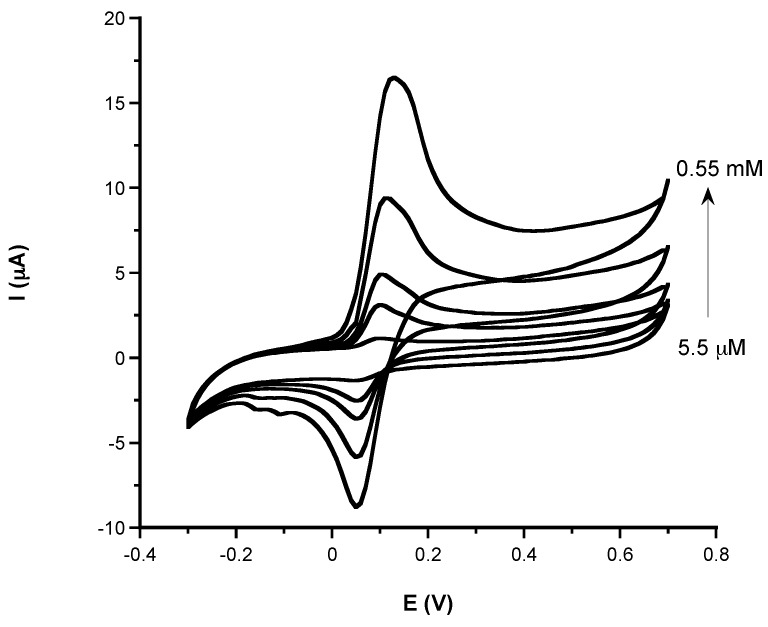
CVs of modified OHT-069 SPE in presence of different CA concentrations: 5.5, 45, 120, 270, and 550 μM, at ν = 10 mV s^−1^.

**Figure 4:**
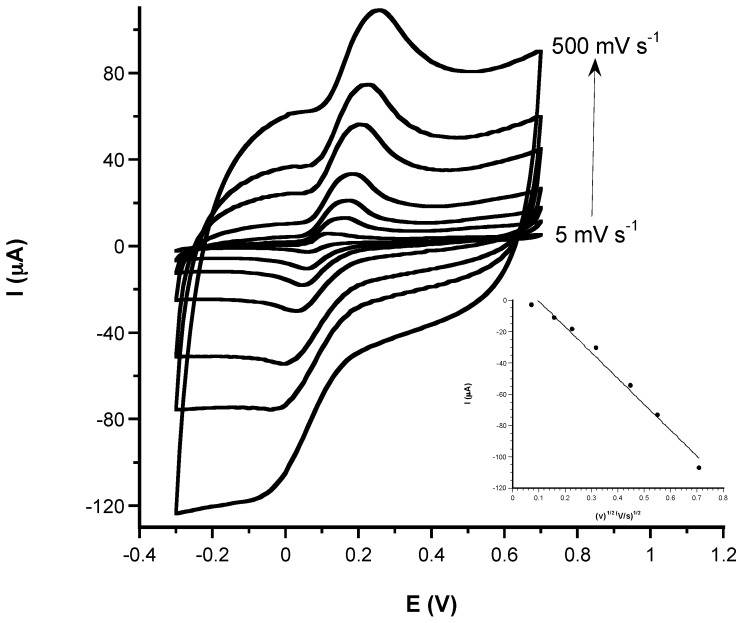
CVs of CA 550 μM using the modified OHT-069 SPE recorded in PBS buffer solution at scan rates of: 5, 25, 50, 100, 200, 300, and 500 mV s^−1^, respectively. Inset: plot of reduction current vs. the square root of scan rate.

The reduction current was proportional with the amount of CA added on the electrode, as illustrated in Figure 3. By increasing CA concentration from 5.5 to 550 µM, the oxidation/reduction peak potential is constant at 0.130 and 0.050 V, respectively. These results suggest that diffusional barriers are not present for the oxidized/reduced CA to/from nanoceria surface. Figure 4 shows the effect of scan rate on the intensity of the oxidation/reduction current. In the inset of Figure 4 is reported the reduction current vs. the square root of scan rate. The current increased proportionally with the square root of scan rate with a correlation coefficient of 0.99 (N = 3) with a slight shift towards negative potentials, suggesting a quasi-reversible process, which is diffusion-controlled.

#### 3.1.2. Analytical Characteristic: Sensitivity, LOD, Linear Range, and Response Time of Phenolic and Non-Phenolic Antioxidants

The CeNPs modified sensors have been tested for the detection of some common antioxidant compounds contained in wines: gallic acid (GA), caffeic acid (CA), quercetin (Q), t-resveratrol (t-R), and ascorbic acid (AA).

**Figure 5:**
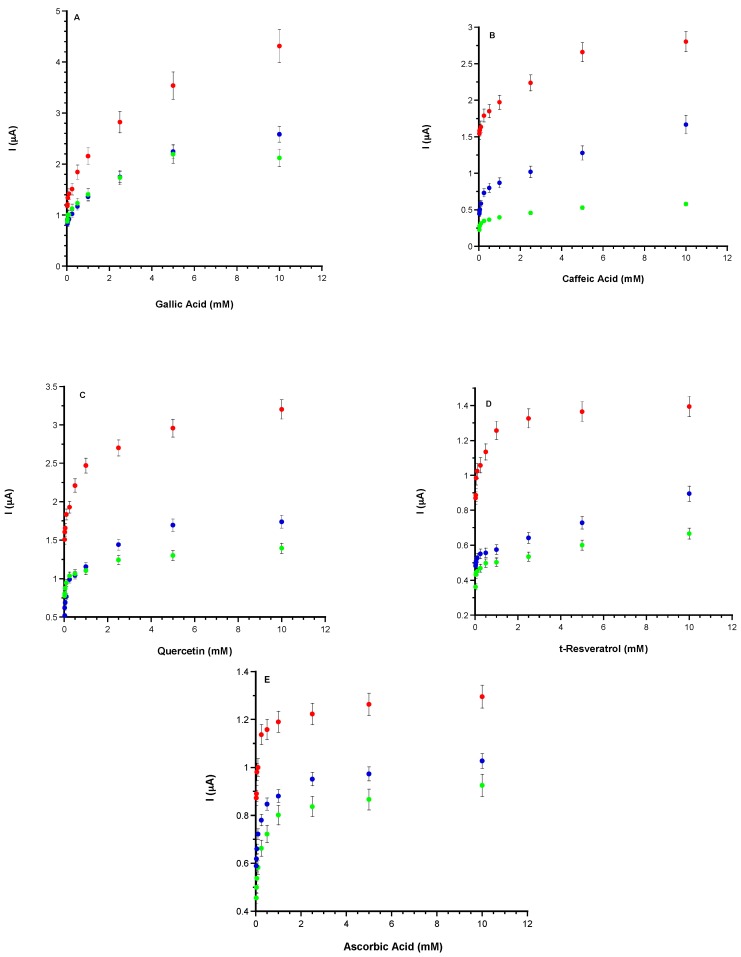
Calibration curves for antioxidant compounds employing SWV technique: gallic acid, GA (**A**), caffeic acid, CA (**B**), quercetin, Q (**C**), t-resveratrol, t-R (**D**), and ascorbic acid, AA (**E**) at different concentrations. The modified SPE-sensors are: OHT-069 (red circle), OHT-102 (blue circle), and OHT-000 (red circle), respectively.

Figure 5 displays the calibration curves of the three different sensors for the selected antioxidant compounds. After the addition of the antioxidant compound a response proportional with the concentration was observed.

The sensitivity of the different sensors towards the same antioxidant was studied and the results reported in Figure 5A–E. The order OHT-069 > OHT-102 > OHT-000 was observed.

The analytical parameters of the antioxidants have been reported by applying the CeNPs modified OHT-069 sensor (Table 2).

### 3.2. Spectrophotometric Characterization

**Scheme 1:**
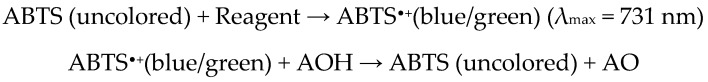
Formation of radical ABTS and its reaction with antioxidants (AOH).

The ABTS-based assay described here involves the direct production of the blue/green ABTS^•+^ chromophore. This has absorption maxima at 731 nm. The addition of antioxidant compounds quenches the colour and produce a decolouration of the solution which is proportional to their amount (Scheme 1). This reaction is rapid and the end point, which is stable, is taken as a measure of the antioxidative efficiency.

The phenolic antioxidant Gallic Acid (GA) was used as standard and the values of real samples expressed as mg L^−1^ of GA.

In Table 3 we have reported a comparison with some enzyme-based biosensors and nanomaterial-based sensors for the determination of some common antioxidants, present in the literature [50,57,87,88,89,90,91,92]. The linear range of our developed sensor is similar and, in some cases, better than ones proposed by other authors. In terms of storage, our sensor is superior respect to the enzyme biosensors because no needs of storage conditions, on the contrary enzyme-based biosensors need of particular conditions (e.g., buffer and low temperature) and one of their drawbacks is protein instability [50,88,90]. Furthermore, in comparison to the nanomaterial CeNP-modified electrode for GA and AA compounds our linear range is broader [57]. Additionally, our LOD value is lower for CA and Q antioxidants [57]. In terms of response time, we have recorded a response time of 30 s. Lastly, these advantages make our sensor as a useful tool for a quick screening to detect antioxidants in beverages sample.

### 3.3. Real Sample Analysis

Real samples (common antioxidants and white/red commercial wines) were tested for their antioxidant activity. The real samples were analysed both by the OHT-069-modified sensor and the ABTS-based assay.

By the first method, the antioxidant activity of the wine sample was reported as mg L^−1^ of GA, obtained by interpolation of the current signal of the sample into the calibration curve of the GA. The wine white sample were diluted 10 times before analysis and the red ones 100.

For comparison purpose, the same samples were also analysed by the spectrophotometric method involving the use of the ABTS to calculate the total antioxidant capacity (TAC).

As reported in Table 4, the data obtained with the two methods are in good agreement despite the electrochemical and the spectrophotometric methods are based on a completely different mechanism. The results relative to white and red wines are in very close agreement with the data obtained with the spectrophotometric test. Finally, we can conclude that the nanoceria modified sensor could be employed as first tool for the determination of TAC in wine samples. It can be considered as a valid alternative to commercially available spectrophotometric kits thanks to several advantages such as ease of use, rapidity, cost-effectiveness, and portability.

## Figures and Tables

**Table 1:** Comparison of the electroactive area (A_e_) before and after CeNPs modification.

Sensor	A_e_^a^ (mm^2^)	A_e_^b^ (mm^2^)	ρ ^a^	ρ ^b^
OHT-000	2.42 ± 0.02	16.82 ± 0.04	0.19	1.34
OHT-102	6.26 ± 0.02	18.74 ± 0.02	0.50	1.49
OHT-069	8.50 ± 0.03	21.93 ± 0.01	0.68	1.74

^a^ Bare sensor; ^b^ modified sensor by using 5 µL CeNPs solution on the electrode surface.

**Table 2:** Analytical characterization of antioxidant compounds by using CeNPs modified OHT-069 sensor by SWV method.

Antioxidant Compound	Linear Range (mM)		Slope (µA mM^−1^)	R	LOD (mM)
GA	LLR	0.025–0.05	6.55	0.990	0.007
	HLR	0.5–5.0	0.57	0.987	0.151
CA	LLR	0.033–0.1	0.793	0.999	0.010
	HLR	0.25–5.0	0.180	0.998	0.083
Q	LLR	0.025–0.1	3.978	0.978	0.009
	HLR	1.0–5.0	0.120	0.994	0.303
t-R	LLR	0.025–0.05	4.791	0.982	0.008
	HLR	0.1–1.0	0.262	0.999	0.033
AA	LLR	0.025–1	1.667	0.999	0.007
	HLR	1.0–5.0	0.180	0.996	0.334

LLR: Low Linear Range; HLR: High Linear Range.

**Table 3:** Comparison of some analytical characteristics for antioxidants detection of some enzyme-based biosensors and nanomaterial-based sensors.

Sensor	Antioxidant Compound	LR (µM)	LOD (µM)	Storage/Response Time	Ref.
Lac/pESm/Pt electrode	CA	10–80	-	-	[87]
Lac/PAP/SWCNTs/SPE	GA	0.53–96	-	10 d at 4 °C/-	[88]
Lac/oxygen electrode	CA	0.10–1.00	0.06	-	[89]
Lac/Fc/SPE		2.0–30.0	1.6	-	
Tyr/BSA/GA/Fc/SPE	GA	30.0–300	57	30 d at 4 °C/-	[90]
	CA	10.6–266	10.5		
ITO/Lac/Tyr/CS/MWCNTs electrode	GA	1.6–8.1	1.5	8 d at 4 °C dry atmosphere/-	[50]
	CA	0.4–7.4	0.3		
POx/Fc/MWCNTs/ MO electrode	CA	0.3–383	0.1	-	[91]
	t-R	0.2–228	0.1		
GC/MWCNTs/PEI/electrode	GA	0.6–12	0.04		
	CA	0.6–12	0.08	-	[92]
	Q	0.3–6	0.03		
CeNPs/C/SPE	GA	2–20	1.5	RT/40 s	[57]
	CA	50–100	15.3		
	Q	20–200	8.6		
	AA	0.5–20	0.4		
CeNPs/MWCNTs-COOH/SPE	GA	25–50	7		**Our work**
	CA	33–100	10		
	Q	25–100	8	RT/30 s	
	t-R	25–50	8		
	AA	25–100	7		

Lac: Laccase; pESm: polyether sulphone membrane; PAP: polyazetidine prepolymer; Fc: ferrocene; Tyr: tyrosinase; CS: chitosan; BSA: bovine serum albumine; GA: glutaraldehyde; PEI: polyethylenimine; POx; peroxidase; MO: mineral oil; C: carbon.

**Table 4:** TAC of several white and red wines assessed in parallel by the nanoceria-based electrochemical assay and by the classic TEAC assay.

Wine Sample	CeNPs-SPE ^a^	ABTS-Based Method ^a^
white 1	204 ± 10	208 ± 17
white 2	183 ± 11	187 ± 9
white 3	212 ± 18	209 ± 15
red 1	2067 ± 207	2078 ± 187
red 2	2340 ± 210	2538 ± 228
red 3	3938 ± 236	4172 ± 292

^a^ Expressed as mg L^−1^ of GA.
